# FGFs/FGFRs-dependent signalling in regulation of steroid hormone receptors – implications for therapy of luminal breast cancer

**DOI:** 10.1186/s13046-019-1236-6

**Published:** 2019-05-29

**Authors:** Dominika Piasecka, Marcin Braun, Kamila Kitowska, Kamil Mieczkowski, Radzislaw Kordek, Rafal Sadej, Hanna Romanska

**Affiliations:** 1 Department of Pathology, Chair of Oncology, Medical University of Lodz, Pomorska 251, 92-213 Lodz, Poland; 2 Department of Molecular Enzymology, Intercollegiate Faculty of Biotechnology, University of Gdansk and Medical University of Gdansk, Debinki 1 Street, 80-211 Gdansk, Poland

**Keywords:** Breast cancer, Estrogen receptor, Progesterone receptor, Tumour microenvironment, Fibroblast growth factor receptors

## Abstract

Stromal stimuli mediated by growth factor receptors, leading to ligand-independent activation of steroid hormone receptors, have long been implicated in development of breast cancer resistance to endocrine therapy. Mutations in fibroblast growth factor receptor (FGFR) genes have been associated with a higher incidence and progression of breast cancer. Increasing evidence suggests that FGFR-mediated interaction between luminal invasive ductal breast carcinoma (IDC) and its microenvironment contributes to the progression to hormone-independence. Therapeutic strategies based on FGFR inhibitors hold promise for overcoming resistance to the ER-targeting treatment. A series of excellent reviews discuss a potential role of FGFR in development of IDC. Here, we provide a concise updated summary of existing literature on FGFR-mediated signalling with an emphasis on an interaction between FGFR and estrogen/progesterone receptors (ER/PR) in IDC. Focusing on the regulatory role of tumour microenvironment in the activity of steroid hormone receptors, we compile the available functional data on FGFRs-mediated signalling, as a fundamental mechanism of luminal IDC progression and failure of anti-ER treatment. We also highlight the translational value of the presented findings and summarize ongoing oncologic clinical trials investigating FGFRs inhibition in interventional studies in breast cancer.

## Background

Invasive ductal breast carcinoma (IDC) is divided into biologically distinct and clinically relevant subgroups on the basis of immunohistochemical status of the estrogen receptor (ER), progesterone receptor (PR), human epidermal growth factor receptor 2 (HER2) and Ki-67 proliferation index [1, 2]. These histopathological subclasses can also be displayed at the molecular level as intrinsic molecular subtypes, i.e. luminal A, luminal B, HER2-enriched or triple-negative IDC [3, 4].

The luminal A subtype represents the majority of IDC cases (50–60%) and is defined as ER+/PR+/HER2−/Ki67^low^ or highly expressing ER-related genes specified in molecular profiles (e.g. PAM50) at the protein or mRNA level, respectively [2, 3, 5, 6]. The luminal B IDC, which represents 10–20% of all cases, is identified with an ER+/PR^low^/HER2+/−/Ki67^high^ phenotype or a type expressing ER-related genes at low-to-moderate levels [3, 5–8]. Among all IDC subtypes, luminal A IDC patients have the best survival rate. This is due to both slow growth of the tumours and availability of ER-targeting agents such as tamoxifen, fulvestrant or aromatase inhibitors [3, 6, 9]. However, despite relatively high efficiency of anti-ER first-line endocrine therapy [9], approximately 45% of women do not respond to tamoxifen (de novo resistance), whereas acquired resistance to the drug develops ultimately in all tamoxifen-receiving patients, posing a serious clinical problem [10]. De novo resistance to hormone therapy is particularly frequent in patients with luminal B IDC [8, 11–13]. Cancer cells of luminal B type are characterised by low or negative expression of PR, which as an ER-regulated gene, is thought to reflect steroid hormone-dependency and thus regarded as a predictor of responsiveness to endocrine therapy. In addition, luminal B cells express a number of ER-independent cell cycle proteins, tyrosine kinase receptors and components of their downstream signalling pathways, which render luminal B IDC partially independent of hormonal stimulation [8, 11]. Poor responsiveness of luminal B tumours to endocrine therapy was confirmed by several studies [12–16]. Investigation into the mechanisms underlying regulation of steroid hormone receptors’ function and development of steroid hormone independence is, therefore, a pursuit of modern oncology.

It has been now well acknowledged that a cross-talk between tumour and its microenvironment (TME – tumour microenvironment) can promote cancer progression and development of resistance to therapy [17–22]. Stromal cells i.e. fibroblasts, immune and inflammatory cells, adipocytes and neuroendocrine cells secrete a wide range of a substances such as growth factors (e.g. FGFs, VEGF, EGF, TGFβ), cytokines and chemokines (e.g. IL-1, IL-6, TNFα) [23]. A number of TME-derived factors have been implicated in mediation of tumour – TME interaction. For example, infiltrating inflammatory cells modulated cell invasiveness by providing a ‘chemotactic escape route’ facilitating migration of cancer cells from the bulk of the tumour [24–26]. In breast cancer, a reciprocal paracrine loop between macrophages and cancer cells, involving EGF, CSF-1, CSF-2 or CCL18, led to the epithelial to mesenchymal transition (EMT), increased cell motility, invasion and metastasis [27, 28]. Cancer-associated fibroblasts (CAFs) are one of the most abundant cellular components of the stroma in various epithelial tumours, including breast carcinoma. Moreover, of all growth factors/cytokines secreted by CAFs, fibroblast growth factors (FGFs) emerged as the most powerful mediators of breast cancer progression, function of steroid hormone receptors and resistance to endocrine therapies [29–35].

## Main text

### Fibroblast growth factor receptors in breast cancer

FGFR family consists of four transmembrane receptors (FGFR1–4) containing intracellular domain with kinase activity [36–38]. There are eighteen known FGFs that bind with a different affinity to one or few members of the FGFR family [38, 39]. The signal from FGF receptors is transduced via Ras-dependent mitogen-activated protein kinase (MAPK), phosphoinositide 3-kinase (PI3K)/AKT or STATs-dependent pathways [40, 41]. In the organogenesis of the mammary gland, FGF/FGFR signalling (especially the FGF10/FGFR2 axis) controls the very early stages of steroid hormone-dependent development of the ducts as well as survival and proliferation of postnatal mammary luminal and basal epithelial cells [42–44]. FGF/FGFR signalling plays a fundamental role in numerous physiological processes and its dysregulation has been associated with several developmental abnormalities and malignancies, including IDC.

Amplification/overexpression of *FGFR1*, *FGFR2* and *FGFR4* was reported as the most frequent genetic aberrancy within the FGFR family in human cancer [38, 45–47]. *FGFR1* is amplified in 8.7% of all breast cancers and this was shown as an independent predictor of overall survival [48]. Amplifications of *FGFR2* and *FGFR4* are rarer, observed in less than 1 and 2.3% of breast cancer patients, respectively [49]. There is a strong evidence for the association between point mutations in *FGFR* genes and breast cancer aggressiveness, metastasis as well as resistance to chemo- and endocrine therapy [50–55]. Moreover, several polymorphisms in *FGFR2, FGFR3* and *FGFR4*, but not *FGFR1* gene, were associated with a high risk of IDC [38, 56–65]. Biological consequences of *FGFR2* polymorphism were confirmed in several meta-analyses. The ten most frequent *FGFR2* polymorphisms (rs1078806, rs11200014, rs1219648, rs2420946, rs2981578, rs2981579, rs2981582, rs3135718, rs10736303, and rs3750817), out of all 23 reported in the literature, were found to be significantly associated with an increased breast cancer risk in a total of 121,740 cases and 198,549 controls recruited for the biggest of the study [15, 57–59, 61–63, 65, 66]. Interestingly, polymorphisms in *FGFR2* (rs2981582, rs1219648, and rs2420946) were characterised by a strong association with the risk of ER-positive but not ER-negative IDC [67, 68]. This was additionally confirmed in the meta-analysis by Wang et al., which involved 288,142 participants of 37 studies [62]. *FGFR4* rs351855 was repeatedly reported to be associated not only with a higher risk of breast cancer, but also with its aggressiveness and resistance to anti-ER treatment [38, 50, 51]. Functional studies revealed that polymorphisms in *FGFRs* most commonly consist of missense mutations, which result in either alteration of the structure of ligand-binding domain or constitutive activation of FGFR kinase domain [38, 50, 51, 53]. The specificity to luminal IDC relates also to the genetic polymorphism of the FGFR ligands. *FGF10* (one of FGFR2 ligands) rs10941679 was associated with a higher risk of luminal IDC and reported to result in overexpression of FGF10 and hyperactivation of the FGFR2 pathway in ER-positive IDC cells [65].

Over the last few years *FGFR* genes have emerged as important players in the pathogenesis of diverse carcinomas, including luminal IDC. This review summarizes for the first time the existing experimental and clinical data on the cross-talk between steroid hormone receptors and the FGFs/FGFRs axis in view of their relevance to ER-targeting therapy.

### ER and PR – signalling pathways and regulation

**Fig. 1:**
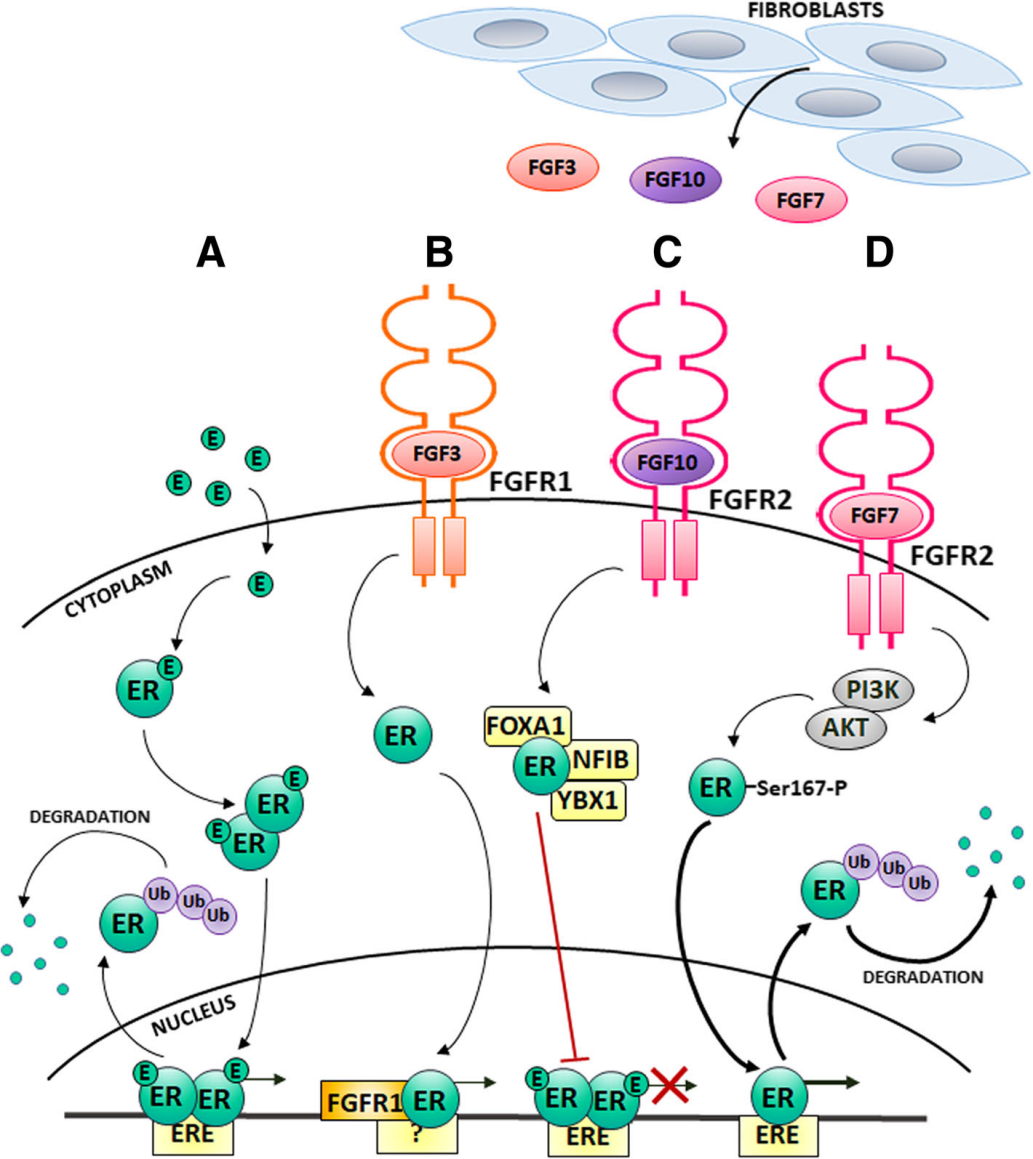
Estrogen receptor (ER) activity in breast cancer - canonical (classical; ligand-mediated) and non-canonical (alternative; ligand-independent) pathways of ER activation. **a** In the canonical model, estrogen binds to ER, which results in receptor dimerization, subsequent translocation to the nucleus and binding to specific genomic sequences i.e. estrogen responsive elements. Activated ER interacts with co-regulators, that modulate target genes expression. This is followed by ER ubiquitination and degradation via a 26-proteasome complex. In the non-canonical pathways (**b**-**d**), activity of ER is regulated in a ligand-independent manner by stimuli (FGFs) from the tumour microenvironment (TME). Binding of FGFs to their cognate receptors, FGFRs, induces FGFRS-triggered signalling, which targets ER. **b** FGF3/FGFR1-triggered signalling leads to induction of ER-FGFR1 complex formation, which binds to unknown genomic sequences and regulates expression of ER-dependent genes. **c** FGF10/FGFR2-activated pathway strengthens the interaction between ER and two transcription factors (NFIB and YBX1), which upon binding to ER-FOXA1 suppress ER-dependent gene expression, **d** FGF7/FGFR2-dependent activation of PI3K/AKT induces ER phosphorylation, enhanced ER transcriptional activity and increased ER degradation. E – estrogen; ER – estrogen receptor; ERE – estrogen responsive element; Ub - ubiquitin

**Fig. 2:**
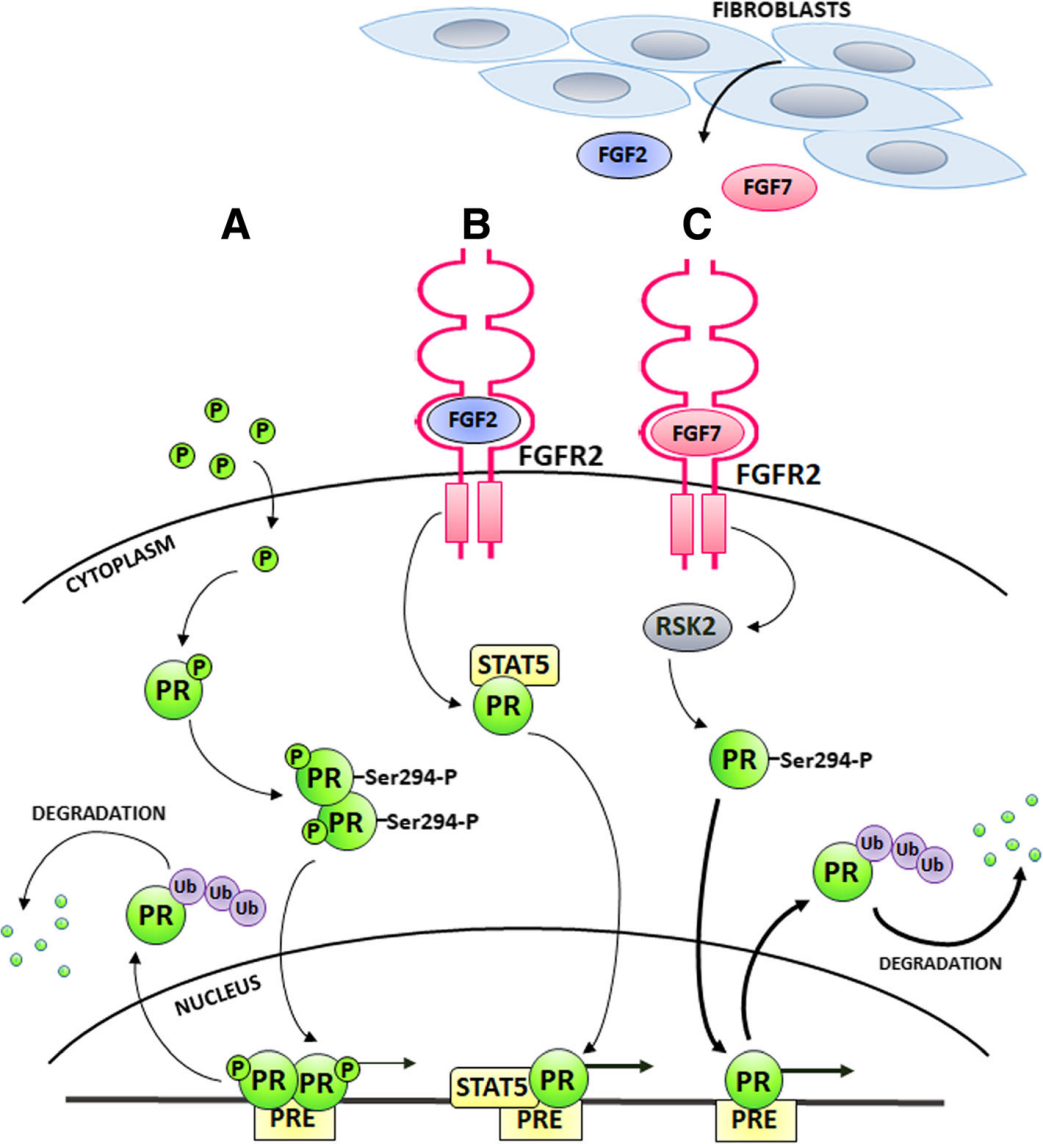
Progesterone receptor activity in breast cancer - canonical (classical; ligand-mediated) and non-canonical (alternative; ligand-independent) pathways of PR activation. **a** In the classical model, progesterone binds to PR, which induces receptor dimerization, translocation to the nucleus and binding to PR specific genomic sequences i.e. progesterone responsive elements. This results in regulation of expression of PR-dependent genes, followed by PR ubiquitination and proteasomal degradation. In the non-canonical pathways (**b**-**c**), PR activation is induced by tyrosine kinases. FGFRs mediate a tumour microenvironment-originated signal (FGFs), which targets PR. **b** FGF2/FGFR2 signalling leads to PR co-localization with STAT5 in a nucleus of cancer cells, which stimulates transcription of PRE-containing genes. **c** FGF7/FGFR2-triggered signalling increases transcriptional activity of PR via RSK2-mediated PR phosphorylation at Ser294 and subsequent PR ubiquitination and degradation in proteasome. P – progesterone; PR – progesterone receptor; PRE – progesterone responsive element, Ub - ubiquitin

**Fig. 3:**
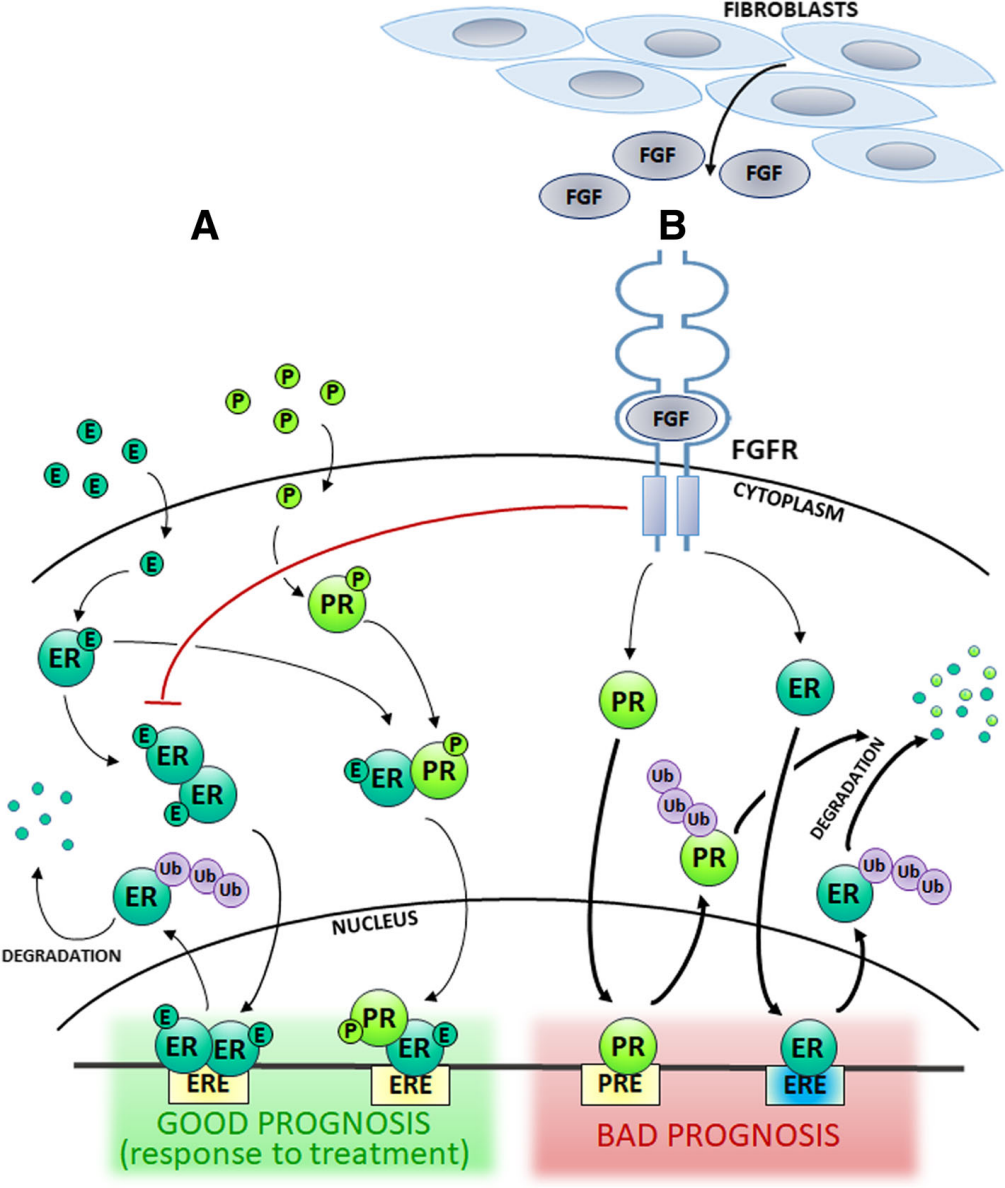
Ligand-dependent and –independent activation of ER/PR – an impact on patient prognosis in luminal IDC. **a** ER is activated in response to estrogen. In addition, progesterone induces PR/ER dimerization and recruits ER away from the classical ER-binding sites to the new PR-directed sites, promoting expression of a gene set associated with GOOD PROGNOSIS. **b** There are two major mechanisms of FGFRs-induced steroid hormone-independent ER/PR regulation, both associated with POOR PROGNOSIS: a FGFRs-triggered shift in ER binding to DNA (ERE, in blue), and FGFRs-dependent rapid activation of ER and PR leading to their subsequent degradation. E – estrogen; ER – estrogen receptor; ERE – estrogen responsive element; P – progesterone; Ub – ubiquitin

Estrogen and progesterone are essential regulators of mammary gland development. Estrogen is strongly involved in a process of ducts’ formation, whereas progesterone promotes growth of the gland lobules [69]. Progesterone and PR work in concert with estrogen and ER to induce expansion of glandular structures during organogenesis of the breast [70]. In addition to their physiological role, both receptors and their cognate ligands have been implicated in development and progression of luminal IDC. ER and PR belong to the nuclear receptors’ family of ligand-activated transcription factors, which regulate genes expression by activation or repression of transcription [71, 72]. Binding of steroid hormones induces receptor dimerization and subsequent conformational changes, which in turn expose nuclear localization signal within the receptor. This is followed by receptor translocation to the nucleus, where it binds to DNA sequences and enhances or silences transcription of target genes. This “classical” pathway of the steroid hormone receptor-mediated signalling is characterised by ER/PR binding to specific genomic sequences i.e. ERE – estrogen and PRE - progesterone responsive element, respectively, and results in the interaction of the receptors with co-regulators to modulate target genes expression (Figs. 1a and 2a). In addition to the conventional steroid hormone-dependent control of ER/PR activity, their reciprocal regulation and cross-talk with various signalling pathways, triggered by growth factor receptors, affect their function. It has been recently shown that, upon progesterone stimulation, PR interacted with ER and recruited it away from the classical ER-binding sites to the new PR-directed locations, resulting in an activation of a set of genes associated with a good clinical outcome (Fig. 3) [73, 74]. As demonstrated for the first time by Denner and co-workers, PR can be phosphorylated and transcriptionally activated independently of progesterone binding [75]. Several reports confirmed growth factor receptors-mediated activation of steroid hormones receptors in the absence of their cognate ligands. It was shown that heregulin treatment of luminal IDC cells resulted in transactivation of PR and this required both functional ErbB2 and MAPK activity [76]. PR was proved to be activated also by other growth factors such as IGF-1 (insulin-like growth factor-1) [77], EGF (epidermal growth factor) [78], FGF2 [79] and FGF7 [80]. Similarly, IGF-1 [81, 82], EGF [83–85] and FGF7 [32] were reported to activate ER in a ligand-independent manner. There is an evidence to suggest that growth factor-mediated ER or PR activation results in steroid hormone receptor phosphorylation followed by its ubiquitination and degradation [86–89]. Moreover, growth factor-dependent activation of MAPK and PI3K (phosphatidylinositol-3-kinase)/AKT leads to ER phosphorylation resulting in cell resistance to tamoxifen [84, 90]. Phosphorylation of PR in response to EGF-triggered signalling can negatively regulate progesterone-induced PR sumoylation [78, 91]. This posttranslational modification by small ubiquitin-like modifier SUMO was previously shown to stabilize PR and inhibit PR transcriptional activity [92]. Consistently, recent studies performed in luminal IDC cells clearly demonstrated that FGF7/FGFR2-triggered phosphorylation of PR at Ser294, followed by PR ubiquitination and receptor degradation via the 26S proteasome pathway [80], contributed to the progression towards a steroid hormone-independent phenotype. Phosphorylated and desumoylated PR is thought to be transcriptionally hyperactive, rapidly turned over and thus difficult to detect (e.g. by routine immunohistochemistry) [78, 91]. If so, it is likely that such a hyperactive and rapidly degraded PR might actually be present in breast tumours clinically classified as PR-low or PR-negative (luminal B IDC). Knutson and co-workers have recently confirmed that phospho-PR Ser294 and elevated expression of a unique phospho-PR genes signature was detected in a substantial subset of phenotypically PR-negative tumours [93].

Taken together, these results demonstrate that signalling triggered by stroma-derived growth factors, which targets ER/PR, might represent a mechanism of IDC progression towards more aggressive steroid hormone-independent phenotype, contributing to the failure of the anti-ER therapies.

### Fibroblast growth factor receptors-dependent signalling and regulation of steroid hormone receptors

A number of studies have unequivocally demonstrated a functional link between FGFRs and steroid hormone receptors. Back in 1998, McLeskey and colleagues, using MCF7-derived cell lines overexpressing FGF1 or FGF4, showed that FGFs were able to replace estrogen as a mitogenic stimulus indispensable for ER-positive tumour growth. Thus, FGFs-dependent signalling bypasses the ER signal transduction pathways and might be responsible for poor response to anti-ER treatments with tamoxifen or fulvestrant [94]. On the other hand, overexpression of FGF8b (a preferential ligand of FGFR1IIIc and FGFR2IIIc splice isoforms as well as FGFR4) in MCF7 luminal IDC cell line led to an increase of anchorage-independent growth and provided an additional growth advantage for cells stimulated with estradiol. FGF8b-overexpression also promoted MMP9 secretion and IDC cell invasion. FGF8b-transfected cells xenografted into nude mice formed faster growing and more densely vascularized tumours [95]. *FGFR1* amplification and overexpression was frequently found in ER-positive/PR-negative IDC tissue, indicating that FGFR1 is strongly associated with worse prognosticating luminal B IDC. Indeed, FGF2/FGFR1-triggered signalling in luminal BCa cell lines with *FGFR1* amplification and overexpression was shown to inhibit ER-directed transcription, which was reflected by suppression of PR expression [96]. It was demonstrated that *FGFR1* is amplified/overexpressed in 43% of ER-positive IDC patients resistant to aromatase inhibitor (letrozole). Interestingly, overexpression of FGFR1 was accompanied by upregulation of *FGF3, FGF4* and *FGF19*. Long-term estrogen-deprivation of CAMA1 luminal BCa cell line, mimicking therapy with letrozole, resulted in increased FGFR1-ER interaction, which required FGFR1 kinase activity. This led to the estrogen-independent induction of ER-regulated genes, which was confirmed by ChIP-seq analysis. Further studies revealed that FGF3 treatment shifted ER and FGFR1 binding to the new chromatin regions, unoccupied in the absence of the FGFRs` ligands (Fig. 1b). Combined inhibition of ER and FGFR with fulvestrant and lucitanib, respectively, abrogated ER or FGFR1 binding to these sites, suggesting that FGF/FGFR pathway modulates ER-DNA interaction. In addition, combination of these inhibitors strongly impaired growth of ER-positive IDC with *FGFR1* amplification. Profiling by qRT-PCR of ER-positive/*FGFR1*-amplified IDCs, deprived of estrogen and treated with FGF3/FGF19, identified a subset of ER-responsive genes, which included *TFF1*, *CCND1*, *THSB1*, *CTGF*, *CCL2* and *EGR3*. In addition, gene-set enrichment analysis (GSEA) from letrozole-treated IDC patients revealed, that ER-related pathways were still active in ER-positive/*FGFR1*-amplified primary tumours. This suggests that association of FGFR1 with ER maintains ligand-independent ER transcription and mediates resistance to estrogen deprivation in ER-positive IDC [97]. *FGFR1* amplification has been shown as an independent negative prognostic factor for disease-free and overall survival exclusively in patients with ER-positive IDC. Chromogenic in situ hybridisation indicated that breast cancer patients with *FGFR1* amplification in the ER-positive group were characterised by lack of PR expression and were at a significantly higher risk for development of distant metastases [48].

FGFR2 activation by FGF10, the most potent FGFR2 ligand in mammary epithelial cells [98], was reported to counteract estrogen-triggered ER-dependent signalling [99]. Further analysis showed that three breast cancer risk SNPs (rs2981578, rs35054928 and rs45631563) in the *FGFR2* locus were responsible for reduced expression of FGFR2, conferred increased estrogen responsiveness and a higher risk of ER-positive IDC [99]. This would suggest that reduced expression of FGFR2 (due to specific polymorphism in *FGFR2* gene) associates with good prognosis. There are data clearly indicating FGFR2 involvement in progression towards ER-negative luminal IDC, a subtype more aggressive and less responsive to the treatment. FGF10/FGFR2 signalling was shown in MCF7 and ZR-75-1 IDC cell lines to strengthen the interaction of ER with two transcription factors, NFIB and YBX1. Binding to the ER-FOXA1 complex, both factors repressed ER target gene expression (Fig. 1c). This suggests that FGFR2 might have a broad effect promoting IDC progression towards estrogen-independent basal-like phenotype and application of FGFR inhibitors could increase tumour sensitivity to anti-ER therapies [100]. Moreover, as demonstrated by our group, treatment of MCF7 and T47D cells with FGF7 or CAFs-conditioned media induced ER ubiquitination and subsequent ER degradation in proteasome. This was mediated by FGFR2-induced PI3K/AKT signalling pathway, which enhanced ER-Ser167 phosphorylation (Fig. 1d). FGFR2-induced ER loss in response to FGF7 and/or CAFs-derived signals in the cell lines was corroborated by an inverse correlation between FGFR2 and ER expression in tissue from IDC patients [32]. A study carried out in a murine model of MPA (medroxyprogesterone acetate - synthetic progestin)-induced mammary carcinoma has revealed that hormone-independent (HI) tumours were characterised by a higher level of FGFR2 expression than their hormone-dependent counterparts. In addition, CAFs isolated from HI tumours were shown to secrete FGF2, which led to phosphorylation of PR (at Ser190 and Ser294) and hormone-independent growth in both HI and luminal IDC (T47D) cells. This effect was abolished by application of PD173074, a FGFRs’ inhibitor, or FGF2 neutralising antibodies [31]. The same group has further demonstrated that FGFR2 co-localized with STAT5 and PR in a nucleus of luminal IDC cells in response to treatment with FGF2 and MPA (Fig. 2b). This nuclear interaction, associated with increased transcription of PRE-containing reporter genes, was also observed in human IDC tissue [79]. In addition, exogenously administered FGF2 was able to mimic MPA, and this effect was reverted by the antiprogestin, RU486 [31, 101]. In our recent study, we found that regulation of activation and turnover of PR was FGFR2-dependent. FGF7/FGFR2-triggered signalling led to PR phosphorylation at Ser294 and subsequent PR ubiquitination and degradation in proteasome. RSK2 kinase was identified as a mediator of FGFR2 action towards PR loss (Fig. 2c). Immunohistochemical analysis of IDC tissue specimens demonstrated that expression of PR inversely correlated with that of an active form of RSK (RSK-P). Patients with RSK-P(+)/PR(−) tumours had a higher risk of recurrence, when compared with the rest of the cohort. These results indicate that the FGFR2-RSK2 signalling pathway activates PR and regulates its turnover, which might contribute to the TME-driven progression of luminal IDC towards steroid hormone-independence [80].

FGFRs are not only the powerful regulators of steroid hormone receptors function but, as shown for FGFR3, they may also act as effective mediators of ER activity. FGFR3 was identified as a key facilitator of ER-driven expansion of breast cancer stem cells (BCSCs). Analyses of antibody-based protein arrays revealed that estrogen treatment induced secretion of FGF family members i.e. FGF2, FGF4, FGF6, FGF7 and FGF9 in MCF7 cell line. Further in vitro and in vivo studies showed that estrogen stimulation led to the expansion of functional BCSCs pool through a paracrine FGF9/FGFR3/Tbx3 signalling. This suggests that FGF9/FGFR3/Tbx3-mediated promotion of BCSCs’ survival and growth might be one of the mechanisms responsible for the failure of treatment, including ER-targeting therapies [52].

These studies demonstrate that in response to stromal stimuli, FGF/FGFR signalling not only regulates steroid hormone receptors turnover, but also determines their transcriptional activity and DNA-binding, which might contribute to IDC progression towards steroid hormone- independence.

### CAFs/FGFs/FGFRs and response to anti-ER treatment

There is growing evidence that regulation of ER and PR function by tumour microenvironment contributes to breast cancer progression. Stromal cells were shown to upregulate aromatase expression and increase estrogen levels in the tumour [102]. An impact of CAFs on response to endocrine therapy has been demonstrated in numerous studies. For example, co-culture of premalignant mammary cells (EIII8, a subclone of MCF10A) or invasive IDC cells (MCF7) with fibroblasts derived from ER/PR-positive tumours enhanced inhibitory effect of tamoxifen on cell growth in 3D cultures, whereas fibroblasts from of ER/PR-negative tumours triggered an opposite effect i.e. promoted acquisition of resistance to tamoxifen [35]. Recent study suggested that effectiveness of tamoxifen and patients` outcome in luminal IDC are determined by CAFs’ phenotype. In the presence of CD146-negative CAFs, MCF7 cells implanted into mice displayed decreased ER expression, diminished sensitivity to estrogen and increased resistance to tamoxifen. Conversely, CD146-positive CAFs led to a sustained ER expression, estrogen-dependent proliferation and sensitivity to tamoxifen [29]. In addition, in ER-positive IDCs, existence of CAFs subpopulation with low level of ERK phosphorylation was associated with worse response of patients to tamoxifen-based therapy. This suggests that a status of ERK phosphorylation in CAFs might be used as a biomarker of efficiency of anti-ER treatment [103].

Being a rich source of FGFs in tumour stroma [31, 79, 104], CAFs influence luminal IDC response to endocrine therapy through FGFs/FGFRs-mediated regulation of steroid hormone receptors. Results of several studies in a murine model of estradiol-dependent breast carcinoma and human ER-positive IDC cell lines demonstrate that CAFs protect cancer cells from tamoxifen-induced cell death via activation of AKT and MAPK pathways, which leads to ER phosphorylation [32, 33, 35]. Both FGF7 and CAF-conditioned medium counteracted tamoxifen-dependent growth inhibition and this involved FGFR2 activity [32]. An association between FGFs/FGFRs axis and resistance to tamoxifen was first demonstrated twenty years ago in MCF7 cells overexpressing FGF1 and FGF4, xenografted into nude mice [105, 106]. This was further confirmed by Turner et al. showing that *FGFR1*-amplified cell lines (MDA-MB-134 and SUM44) displayed resistance to tamoxifen. FGF2/FGFR1 signalling was suggested to overcome tamoxifen-induced growth arrest and apoptosis, which has been linked with high MAPK and AKT activity as well as increased level of cyclin D1. Poor prognosis of patients with FGFR1-overexpressing tumours subjected to adjuvant tamoxifen-based therapy verified results of experimental studies. FGFR1 signalling was shown to suppress PR expression in vitro and this was confirmed by demonstration of an inverse correlation between FGFR1 and PR in human breast cancer tissue [96]. A novel mechanism of resistance to endocrine therapies in ER-positive IDC with *FGFR1* amplification was proposed by Formisano et al. Long-term estrogen-deprivation of FGFR1-overexpressing CAMA1 luminal IDC cell line, mimicking effect of aromatase inhibitors, enhanced FGFR1 interaction with ER, leading to the induction of expression of ER-dependent genes. Simultaneous inhibition of FGFR1 and ER (with lucitanib and fulvestrant) supressed cell growth in vitro and in a PDX (patient derived xenograft) model more potently than when the drugs were administered separately. This implies that patients with endocrine resistant ER-positive/*FGFR1*-amplified tumours may benefit from the treatment with combination of ER and FGFR antagonists [97]. This finding was supported by genomic profiling of 155 early ER-positive IDCs exposed to short-term estrogen suppression with letrozole, which identified amplification of *FGFR1* and *CCND1* (cyclin D1 gene) as a likely mechanism of resistance to the treatment. *FGFR1/CCND1* co-amplification led to a greater enrichment of cell cycle genes than enhancement caused by single amplifications, which is consistent with activation of alternative mechanisms of escape from canonical cell cycle control. Furthermore, combined inhibition of FGFR1 and CDK4/6 in CAMA1 cell line abolished anti-estrogen resistance suggesting that an interaction between FGFR1 and cyclin D1 might drive estrogen-independent proliferation in co-amplified tumours [107]. FGFR2-dependent signalling was proved to counteract negative effect of tamoxifen on T47D and MCF7 cell growth with molecular mechanism involving PI3K/AKT pathway and regulation of Bcl-2 expression [32]. Resistance to tamoxifen has also been associated with increased expression of *FGFR3*. The FGF1/FGFR3 axis conferred resistance to both tamoxifen and fulvestrant in an ER-independent manner (no activation of ER was observed) in MCF7 cell line. The mechanism of FGFR3-promoted proliferation of tamoxifen resistant cells relied on activation of PLCγ/PI3K and MAPK pathways, however, inhibition of only the former resulted in reversal of the tamoxifen-resistant phenotype [108]. An elevated level of FGFR4 mRNA was reported as an independent predictor of little clinical benefit and shorter progression-free survival in IDC patients treated with tamoxifen [109].

In summary, presented studies demonstrate that microenvironmental stimuli from specific CAFs subpopulations may act as a dual-face regulator of resistance to endocrine therapy. Co-operation of hormone receptors with FGF/FGFR-triggered signalling pathway might be an important mediator of steroid hormone independence.

### Therapeutic targeting and future perspectives

The described findings demonstrate that steroid hormone-independent shift in ER binding to DNA or induction of rapid ER/PR activation triggered by FGFR are followed by ER and PR degradation (Fig. 3). This implicates that FGF/FGFR signalling pathway acts as an essential regulator of steroid hormone receptors activity. It mediates resistance to endocrine therapy induced by microenvironmental stimuli. The FGF/FGFR axis is, therefore, a promising target for therapy of luminal IDC [38, 110, 111].

The established strategies for inhibition of the FGFR/FGF pathway fall into three main categories/classes: 1) non-selective tyrosine kinase inhibitors, which act against the intracellular domains of not only FGFRs, but also VEGFRs or PDGFRs; 2) selective inhibitors of FGFRs, which target all FGFR1–3 (due to strong similarity of structure within the receptor family, no selective inhibitors for individual FGFRs are available) or FGFR4; 3) monoclonal antibodies that either block FGFRs or entrap their ligands (reviewed in 38, 110). According to clinicaltrials.gov, there have been 179 completed or ongoing oncologic clinical trials investigating FGFs/FGFRs inhibition in interventional studies, eighteen of which concern breast cancer patients (phase I and II, NCT numbers: NCT03238196, NCT00958971, NCT02053636, NCT01202591, NCT02202746, NCT03344536, NCT01791985, NCT02619162, NCT01795768, NCT02511847, NCT02915172, NCT01594177, NCT02465060, NCT02052778, NCT01928459, NCT03514121, NCT02393248, NCT03583125).

Dovitinib (TKI258, Novartis) is an example of a non-selective inhibitor of FGFR family showing high potency for c-KIT, CSF-1, VEGFR and PDGFR which has been tested in six Phase I/II clinical trials involving advanced breast cancer patients [38, 112]. Musolino et al. showed cautiously promising efficacy - complete and partial responses - after dovitinib administration in advanced, hormone-resistant ER-positive, HER2-negative, FGF-amplified breast cancer patients [113]. Cheng et al. reported an almost complete response (including brain lesions) to pazopanib – another multikinase inhibitor - in a patient with hormone-resistant ER-positive, HER2-negative and FGFR1-amplified IDC [114]. Lenvatinib (E7080, Eisai) is another non-selective RTK inhibitor, which targets FGFR1–4, VGFR1, PDGFR, RET and KIT and was reported as a promising drug for aggressive, triple-negative breast cancer patients [115]. AZD4547, NVP-BGJ398 and JNJ-42756493 belong to the second class of selective FGFR inhibitors, and are currently under a Phase I/II clinical trial to evaluate their activity in patients with amplified FGFR1 and FGFR2 breast, squamous lung and stomach cancers [38, 116]. In breast cancer, both NVP-BGJ398 and AZD4547 showed promising results in studies by Smyth et al. and Nogova et al., where patients with advanced breast cancers responded partially to their application [117, 118]. Monoclonal antibodies are the third major class of FGF/FGFR-targeting agents. Numerous antibodies have been developed, however the knowledge about their clinical potential is limited to only a few (reviewed in 38, 111). FPA144 and MFGR1877S, monoclonal antibodies to FGFR3 and FGFR2, respectively, and FP-1039, a FGF2 trap, showed promising activities with acceptable toxicity in advanced solid tumours [119–121]. Clinical trials of anti-FGFR monoclonal antibodies specifically in breast cancer patients have not been reported, yet.

Anti-FGF/FGFR agents are tested in clinical trials either alone or, more commonly, in combination with other therapies (with standard therapies, immunotherapy or other targeted therapies) [111]. In luminal breast cancer, the most promising possibility is to combine FGFR inhibitors with anti-ER therapies. Results reported by Musolino et al. encouraged development of new trials testing such combinations (fulvestrant, palbociclib and erdafitinib in NCT03238196, AZD4547 and fulvestrant NCT01202591, Debio 1347 and fulvestrant in NCT03344536, AZD4547 and Anastrozole or Letrozole in NCT01791985, nintedanib and letrozole in NCT02619162) [113]. No multiple therapies combining FGFR inhibitors with immunotherapy (unlike in HER2-positive breast cancer - PA150–001 with pembrolizumab (NCT03514121) or afatinib together with trastuzumab and chemotherapy (NCT01594177)) have been developed yet.

In spite of an undisputed role of FGF/FGFR signalling in cancer progression, potential benefits of their clinical use are accompanied with downsides such as side effects. These include hyperphosphatemia, skin and eye dryness, keratopathy, asymptomatic retinal pigment epithelial detachment, hypertension, proteinuria, cardiac, vascular or liver impairment, diarrhoea and fatigue nausea [38, 111]. In addition, as pertinent to all targeted therapies, various hurdles, particularly those related to tumour heterogeneity (an existence of only a subset of sensitive/responsive cells), acquired resistance, identification of predictive markers for appropriate selection of patients, need to be overcome before their routine implementation in clinic is granted. Results from early clinical trials hold promise for therapeutic efficiency of anti-FGF/FGFR agents as a complementary strategy in ER-positive breast cancer. Further functional studies are required so their use can bring lasting therapeutic benefit.

## Conclusion

Prognosis of luminal IDC largely depends on cell response to endocrine therapy. This relies on expression of hormone receptors (ER/PR) and `addiction` of cancer cells to steroid hormones. Functional studies and clinical analyses provide ample evidence that activity of ER and PR is affected by stroma-derived stimuli mediated FGFRs. FGFRs-triggered signalling can lead to emergence of steroid hormone independence and progression towards ER/PR-negative IDC. These findings open new avenues for development of new therapeutic strategies based on FGFR inhibitors, likely to overcome resistance to commonly applied ER-targeting regimens.

## Abbreviations

BCa: Breast carcinoma
BCSC: Breast cancer stem cells
CAFs: Cancer-associated fibroblasts
ER: Estrogen receptor
ERE: Estrogen responsive element
FGF: Fibroblast growth factor
FGFR: Fibroblast growth factor receptor
HI: Hormone independence
IDC: Invasive ductal carcinoma
MAPK: Mitogen-activated protein kinase
PI3K: Phosphoinositide 3-kinase
PR: Progesterone receptor
PRE: Progesterone responsive element
TME: Tumour microenvironment
